# Evaluation of the Fermentation Profiles and Quality Attributes of Yogurts Made from Cow, Goat, and Mixed Milk

**DOI:** 10.3390/foods15020314

**Published:** 2026-01-15

**Authors:** Agnieszka Jankowska, Maria Wachowska, Aneta Dąbrowska, Marika Bielecka, Józef Warechowski, Aleksandra Potaś

**Affiliations:** 1 Department of Process Engineering, Equipment and Food Biotechnology, Faculty of Food Science, University of Warmia and Mazury, Oczapowskiego 7, 10-719 Olsztyn, Poland; mari@uwm.edu.pl (M.W.); jozefw@uwm.edu.pl (J.W.); aleksandra.potas@uwm.edu.pl (A.P.); 2 Department of Dairy Science and Quality Management, Faculty of Food Science, University of Warmia and Mazury, Oczapowskiego 7, 10-719 Olsztyn, Poland; anetazj@uwm.edu.pl (A.D.); marika.kowalska@uwm.edu.pl (M.B.)

**Keywords:** cow milk, goat milk, yogurt, yogurt starter culture, fermentation pattern, texture, sensory analysis, volatile organic compounds

## Abstract

The use of mixed cow–goat milk in yogurt production allows for balancing fermentation performance, textural properties and sensory attributes of the products. This study evaluated the fermentation behavior and physicochemical, microbiological, textural, and color properties of yogurts produced from cow milk (A), goat milk (E), and mixed cow–goat milk at volume ratios (*v*/*v*) of 75:25 (B), 50:50 (C), and 25:75 (D). Volatile organic compounds (VOCs) were analyzed in milk before fermentation and in yogurts after production and during two weeks of refrigerated storage. Sensory attributes were assessed after two weeks of storage. An increase in the proportion of goat milk in the milk blend shortened the fermentation time, whereas a higher proportion of cow milk enhanced the survival of lactic acid bacteria (LAB), improved water-holding capacity (WHC), strengthened textural properties, and reduced syneresis. Yogurts with higher proportions of goat milk exhibited increased lightness and whiteness. Milk type influenced chemical composition, with higher fat content and lower lactose content observed in goat milk yogurts. A higher proportion of goat milk in the milk blend promoted the formation of methyl ketones and aldehydes associated with a characteristic fatty aroma. Among the mixed-milk yogurts, the sample containing 25% goat milk (D) achieved the highest sensory acceptance. The study demonstrated that cow–goat mixed-milk yogurts represented a balanced compromise between textural stability, microbial viability, and sensory acceptance.

## 1. Introduction

Goat milk products play a crucial role in the socioeconomic development of many countries, and their importance extends beyond low-income economies. Goat milk is characterized by outstanding nutritional quality and technological properties, which make it a valuable raw material for direct consumption and for processing into value-added products [1]. At present, global goat milk production is estimated at 20–21 million tons per year. More than half of that output comes from Asia, and substantial quantities are also produced in Africa and Europe [2]. Goat milk production has more than doubled in recent decades and is expected to increase by another 53% by 2030 [3]. Goat milk is thermally preserved and used in the production of cheese, tvorog, yogurt, infant formulas, and ice cream. Surplus production is processed into milk powder [4].

Cow milk and goat milk differ mainly in the proportions of various types of casein (including αs1-casein, αs2-casein, κ-casein, and β-casein), as well as the structure and size of milk fat globules and casein micelles [5]. Casein micelles in goat milk are characterized by a higher degree of dispersion, greater mineralization, and lower hydration than those in cow milk [6,7]. In addition, goat milk contains less αs1-casein, and the proportion of this protein differs among goat breeds [5]. Goat milk also has a higher proportion of smaller milk fat globules [8] than cow milk. These differences can lead to variations in milk behavior during gelling and curd formation, thus affecting the final quality of goat milk products. Goat milk yogurt differs from cow milk yogurt in several key properties, including the firmness of the curd which is softer and less viscous than in cow milk yogurt [9]. In addition, goat milk yogurt is more susceptible to syneresis during storage, and it is characterized by lower gel strength and a more intense taste, compared with cow milk yogurt [1].

Broader use of goat milk can help diversify the dairy market by supporting the development of new value-added fermented products with properties distinct from those of cow milk. Recent years have witnessed growing consumer interest in fermented dairy products, including ripened cheese, tvorog, dairy beverages, and yogurt made from mixed cow–goat milk. These blends combine the nutritional value and functional properties of goat milk with the desirable sensory attributes and technological properties of cow milk [10,11]. The addition of cow milk can improve the texture and consistency of goat milk yogurt by increasing its viscosity and gel stability. In turn, the addition of goat milk contributes to a more balanced fatty acid profile, enhances digestibility, and increases the content of medium-chain fatty acids and bioactive peptides [12,13].

Goat milk is also blended with cow milk to moderate the intense flavor of goat products, thereby increasing their consumer appeal [14]. A study of yogurts containing different proportions of goat milk (25% to 75%) revealed that these products exhibited intermediate rheological and sensory attributes between cow milk and goat milk products, while maintaining high microbial stability [15]. The use of mixed cow–goat milk can be an effective strategy for managing seasonal production surpluses in the dairy industry, while supporting the development of products with diverse nutritional and sensory profiles that meet the growing consumer demand for alternative sources of milk protein.

A number of studies have examined yogurts produced from cow milk, goat milk, and their blends. However, existing research often focuses on specific quality attributes, such as basic physicochemical properties or sensory evaluation. Comparative analyses of the complete fermentation behavior, including acidification profiles, texture parameters, microbiological stability, and volatile organic compound formation, remain limited. Only a few studies have concurrently assessed yogurt quality immediately after production and during refrigerated storage. Therefore, a comprehensive and time-dependent evaluation of yogurts produced from cow milk, goat milk, and their blends is needed.

The aim of this study was to evaluate the effects of cow milk, goat milk, and their mixtures on the fermentation behavior, physicochemical, microbiological, textural, and sensory properties of yogurts.

## 2. Materials and Methods

### 2.1. Milk Preparation and Yogurt Production

The experiment involved commercial goat milk powder (fat—29%, including 19% saturated fatty acids, total protein—26%, carbohydrates—32%, according to the manufacturer’s declaration—Agro-Danmis Gramowscy, Bukowiec, Poland) and cow milk powder (fat—26%, total protein—26%, according to the manufacturer’s declaration—Polmlek, Raciąż, Poland). Reconstituted milk was prepared by dissolving 15 g of milk powder in sterile water (121 °C, for 20 min) to a final volume of 100 mL. The prepared milk was stored overnight at a temperature of 4 °C and then combined at different volume ratios (*v*/*v*) to obtain raw materials with the following composition:

A—100% cow milk
B—75% cow milk + 25% goat milk
C—50% cow milk + 50% goat milk
D—25% cow milk + 75% goat milk
E—100% goat milk

To produce yogurt, milk was heated to 43 °C, inoculated with a freeze-dried starter culture YC-X16 (*Lactobacillus delbrueckii* ssp. *bulgaricus*, *Streptococcus thermophilus*; Novonesis, Czosnów, Poland), and fermented at 43 °C to pH 4.6. Yogurts were allowed to mature for three days at refrigeration temperature and were then stored at 4 °C for two weeks.

### 2.2. Chemical Composition

The proximate chemical composition of milks (A–E) and yogurts (A1–E1) was determined by Fourier-transform infrared (FTIR) spectroscopy using the MilkoScan™ FT1200 apparatus (Foss, Hillerød, Denmark). The content of fat, total protein, lactose, and dry matter was determined in milk before fermentation and in yogurt after production. The MilkoScan™ FT1200, which is routinely used for compositional analysis of milk and fermented dairy products, was calibrated according to the manufacturer’s instructions.

### 2.3. Kinetics of Milk Acidification

During fermentation, the active acidity (pH) of milk was measured with a multi-channel pH/pC/mV multiplexer (Cerko, Gdynia, Poland) equipped with ERH-13-6 electrodes (Hydromet, Gliwice, Poland). The electrodes were immersed in milk immediately after starter culture addition. Changes in pH were recorded every five minutes in the Cerko Lab System program until pH reached 4.6.

### 2.4. Active Acidity (pH)

The pH of non-fermented milk and yogurts was measured with a CP 505 pH-meter (Elmetron, Zabrze, Poland) equipped with an IJ-44C IONODE electrode and calibrated with standard solutions with pH 4.0 and 7.0 (Merck, Warsaw, Poland). The pH of yogurts was determined after production (A1–E1) and after two weeks of refrigerated storage (A3–E3).

### 2.5. Microbiological Analysis

The plate count method was used to enumerate starter bacteria (*Lb. delbrueckii* ssp. *bulgaricus* and *S. thermophilus*) and coliforms in yogurts after production and after two weeks of refrigerated storage. Aliquots of 1 mL were dissolved in 9 mL of sterile saline solution (NaCl 0.9%). Coliform bacteria were enumerated at 30 °C on Violet Red Bile Glucose (pH 7.4; VRBG-agar; Merck/Sigma-Aldrich, Darmstadt, Germany) according to standard microbiological procedures. Starter cultures were enumerated on selective media according to IDF standards (2003). *Lactobacillus delbrueckii* subsp. *bulgaricus* was isolated on MRS agar (pH 5.4; Merck/Sigma-Aldrich, Darmstadt, Germany) by microaerophilic incubation at a temperature of 37 °C for 72 h. *Streptococcus thermophilus* was isolated on M17 agar (pH 7.2; Merck/Sigma-Aldrich, Darmstadt, Germany) by aerobic incubation at 37 °C for 48 h. Microbial counts were expressed as log_10_ of colony-forming units (CFU/mL of yogurt).

### 2.6. Color Analysis

Color was measured using a CM-3500d spectrophotometer (Konica Minolta, Tokyo, Japan) in the CIE Lab* system with illuminant D65, 10° observer, d/8° measurement geometry, and 8 mm aperture. Before the analysis, the device was calibrated with a white tile (L* = 96.79; a* = –0.08; b* = –0.16) and a black trap (L* = 0.02; a* = –0.03; b* = –0.01). The results were analyzed in the CM-S100w Spectra Magic NX program. Color parameters L*, a*, and b*, chroma (C*), and the whiteness index (WI) were calculated.

### 2.7. Determination of Water-Holding Capacity

The water-holding capacity (WHC) of the samples was determined using a modified centrifugation method. A yogurt sample (20 g) was centrifuged at room temperature for 15 min (10,700 g) in a 5804 R centrifuge (Eppendorf, Hamburg, Germany). The supernatant was weighed, and WHC was calculated as the percentage of pellet weight in the total sample weight. The WHC of yogurts was determined after production (A1–E1) and after two weeks of refrigerated storage (A3–E3).

### 2.8. Texture Analysis

The texture of yogurt samples was evaluated using a Brookfield CT3 texture analyzer (Brookfield Engineering Labs. Inc., Middleborough, MA, USA) during a compression test involving a TA11/1000 cylindrical probe with a diameter of 25.4 mm and a height of 35 mm. During the test, the sample was placed in a cylindrical measurement container with an internal diameter of 50 mm, and the probe was pressed into the sample to 80% of the sample depth. Measurement container was filled with 75–80 mL of yogurt. The following analyzer settings were used during the test: pretest speed—1.00 mm/s, test speed—1.0 mm/s, hold time—0 s, return speed—1.0 mm/s, number of cycles—1, trigger load—0.09 N.

The following parameters were calculated after each test: firmness (maximum force in the positive region of the first compression cycle), consistency (area under the curve of the compression cycle), and cohesiveness (maximum force in the negative region during probe return). The textural properties of yogurts were determined after production (A1–E1) and after two weeks of refrigerated storage (A3–E3). The above parameters were calculated in the TexturePro CT V1.9 Build 35 program. The measurements were conducted in triplicate (three independent samples from each experiment), and the results were expressed as means with standard deviation.

### 2.9. Analysis of Volatile Organic Compounds

Volatile organic compounds (VOCs) in milk and yogurt samples were analyzed using a GC–IMS spectrometer (FlavorSpec, Gesellschaft für Analytische Sensorsysteme GmbH, Dortmund, Germany) equipped with an autosampler. In each analysis, a 5 g sample was placed in 10 mL headspace vials. The samples were incubated at a temperature of 60 °C for 10 min to enrich the vapor phase above the liquid in volatile compounds. Next, 500 µL of the headspace above the sample was automatically introduced into the injector operating in splitless mode at a temperature of 80 °C.

Chromatographic separation was conducted using an MXT-5 column (15 m × 0.32 mm × 0.5 µm) with nitrogen (99.99%) as the carrier gas. The following flow program was applied: 2 mL/min from 0 to 10 min, a linear increase to 10 mL/min from 10 to 15 min, and an increase to 100 mL/min from 15 to 40 min. Column temperature was maintained at 40 °C. The separated compounds were directed to the IMS ionization chamber, where ionization was carried out in positive mode using a radioactive ^3^H source (370 MBq). Ions migrated through the drift tube (9.8 cm, 5 kV, 45 °C) under a drift gas flow rate of 150 mL/min.

Compounds were identified based on retention indices (RI) and ion drift times using the GC×IMS Library Search software(ver. 0.4.03). Retention indices were calculated with C4–C9 n-ketones (Anchem, Warsaw, Poland). Signal matching was performed using Kovats retention indices (NITS) and drift time. Data were analyzed in the VOCal program (0.4.03 rev300) with Gallery Plot, Reporter, and 3D Topographic View modules.

### 2.10. Sensory Analysis

The sensory analysis of yogurts made from cow milk, goat milk, and mixed cow–goat milk was conducted two weeks after production using the profiling method EN ISO 13299:2016-05E [16]. Sensory attributes were evaluated on a seven-point scale, where one point denotes the absence of the analyzed attribute, and seven points denote very high intensity of the analyzed attribute. The sensory panel was composed of eight researchers who had many years of experience in evaluating dairy products and whose sensory sensitivity had been validated according to EN ISO 8586:2014–03 [17]. Yogurts were randomly coded and assessed in a sensory analysis laboratory for the intensity of 15 sensory attributes and for overall acceptability. Polish legislation does not require the approval from a bioethics committee in case of sensory evaluation studies involving food products.

### 2.11. Statistical Analysis

The results of the sensory analysis of yogurts, the proximate chemical analysis of milk and yogurt, and the milk color analysis were processed by one-way analysis of variance (ANOVA). The results of color, pH, microbiological, and texture analyses were processed by two-way ANOVA. Next, Fisher’s LSD test was conducted. All results were processed in Statistica 13.5 PL software (Statsoft, Krakow, Poland) at a significance level of 0.05.

## 3. Results

### 3.1. Chemical Composition of Milk and Yogurts

Dry matter content ranged from 15.40% (A) to 14.95% (E) in milk, and from 15.31% (A1) to 14.74% (E1) in yogurts (Table 1). The observed decrease in dry matter content could be linked to the lower casein content of goat milk [14,18]. Bulgaru et al. [15] and Miocinovic et al. [19] found that goat milk products contain less dry matter and are more susceptible to syneresis, which was confirmed in the present study. Lower dry matter content affects texture and color and decreases the WHC of yogurt curd.

The fat content of milk increased with a rise in the proportion of goat milk in the raw milk blend—from 4.29% in cow milk (A) to 4.55% in goat milk (E). Similar values (4.21–4.48%) were noted in yogurts (A1–E1), which indicates that fermentation had no significant effect on fat content (*p* > 0.05). Goat milk is characterized by higher fat content and smaller milk fat globules, which are more readily emulsified and more stable in the colloidal system [1,14,20]. In addition, a higher content of medium-chain fatty acids (C6:0–C10:0) in goat milk than in cow milk [10] may induce differences in the consistency and taste of yogurts, accompanied by their lower firmness and higher acidity [21].

Protein content was determined at 4.19% in cow milk (A) and was somewhat lower at 4.14% in goat milk (E). A slight decrease in these values (to 4.15–4.07%) was observed after fermentation, which could be attributed to protein degradation by the proteolytic enzymes of lactic acid bacteria (LAB) [22]. These results corroborate published data indicating that goat milk contains less κ-casein and more β-casein, which leads to the formation of gels with looser structure and greater susceptibility to syneresis [18,19]. Differences in protein composition significantly affect the texture and stability of fermented products made from goat milk.

The lactose content of the milk blend decreased with an increase in the proportion of goat milk—from 6.06% in sample A to 5.69% in sample E. In yogurts (A1–E1), these values were even lower after fermentation (5.69–5.13%) as lactose was fermented into lactic acid by *S. thermophilus* and *Lb. delbrueckii* ssp. *bulgaricus* bacteria. A similar decrease in lactose content after fermentation was reported by Mituniewicz-Małek et al. [23], who found that the decline in lactose levels is determined by the activity of starter strains and the buffering capacity of milk. In the current study, the decrease in lactose content was more pronounced in yogurts containing goat milk, which could be attributed to a more rapid decrease in pH and more intense fermentation. The chemical composition of both milk and yogurt was influenced by the proportion of goat milk. These results are consistent with the literature data, which indicate that differences in the fat and protein profiles of goat milk affect not only the nutritional value, but also the technological properties and sensory attributes of the produced yogurts.

### 3.2. Acidification Profile and Bacterial Counts

**Figure 1:**
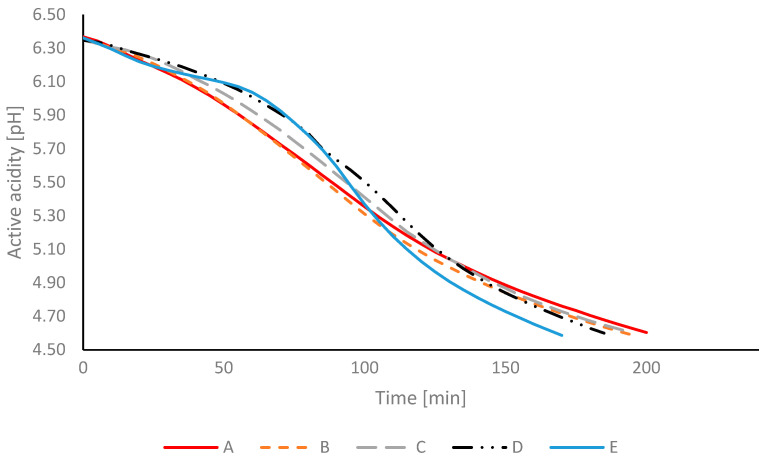
Milk acidification curve (A—100% cow milk; B—75% cow milk + 25% goat milk; C—50% cow milk + 50% goat milk; D—25% cow milk + 75% goat milk; E—100% goat milk) during fermentation with a starter culture (YC-X16) at a temperature of 43 °C (n = 2).

Changes in the pH of cow milk (A), goat milk (E), and cow–goat milk blends (B-D) during fermentation are presented in Figure 1. Initial pH values ranged from 6.35 to 6.37, and they were slightly lower than typical values reported for fresh cow and goat milk. This could result from the processing steps involved in manufacturing milk powder (heat treatment and concentration), which can shift the mineral equilibrium (e.g., calcium phosphate association/precipitation and casein–mineral interactions) and thereby affect buffering capacity and acidity [24,25].

The type of milk influenced fermentation kinetics. A greater increase in acidity was observed in yogurts made from goat milk, whereas in yogurts prepared from cow milk, the decline in pH proceeded at a slower rate. A linear relationship was noted in yogurts made from mixed cow–goat milk: the higher the proportion of cow milk, the slower the acidification kinetics and the longer the fermentation time.

The counts of both LAB species were highest in yogurts made from cow milk and decreased with a rise in the proportion of goat milk (Table 2). The counts of *Lb. delbrueckii* ssp. *bulgaricus* ranged from 7.24 log CFU/mL (A1) to 6.7 log CFU/mL (E1) directly after production and decreased to 6.67–6.01 log CFU/mL (A2-E2) after two weeks of storage. The counts of *S. thermophilus* decreased from 8.79 to 8.59 log CFU/mL directly after production to 8.44–8.00 log CFU/mL after two weeks of storage. In each group of samples, bacterial counts decreased significantly (*p* < 0.05) during storage, but *S. thermophilus* was more stable than *Lb. bulgaricus*. Coliforms bacteria were not identified in any of the samples directly after production or after storage.

The ANOVA also confirmed that bacterial survival was significantly influenced (*p* < 0.05) by milk type. In yogurts with higher proportions of goat milk, the number of CFUs clearly decreased both directly after production and after storage. A similar relationship was observed by Dimitrellou et al. [21] and El-Bannan et al. [26], who found that goat milk yogurts were characterized by lower *Lb. bulgaricus* counts than cow milk yogurts despite similar fermentation conditions. The cited authors attributed their findings to the lower casein content and lower buffering capacity of goat milk, which contribute to a more rapid decrease in pH and faster inhibition of bacterial growth.

Similar results were reported by Mituniewicz-Małek et al. [27], who found that starter culture counts in organic yogurts made from goat and cow milk exceeded 8 log CFU/g for only several days, after which they gradually declined. The survival of LAB during storage varied. The counts of *S. thermophilus* were approximately 1 log CFU/mL higher than *Lb. bulgaricus* counts, which confirms that streptococci are more resistant to low pH and refrigeration [22,28]. According to the cited authors, *S. thermophilus* populations are more stable because they can utilize the products of *Lb. bulgaricus* autolysis and are metabolically better adapted to more acidic environments.

Differences in the composition of goat milk and cow milk, particularly the content of whey proteins, free amino acids, and fatty acids, can affect the environment for bacterial growth [29,30]. In goat milk, the higher concentrations of medium-chain fatty acids and lower buffering capacity promote more rapid acidification, which can compromise the survival of LAB during storage. This observation was confirmed by Yang et al. [31] who reported more extensive changes in the composition of organic compounds in goat milk yogurts after two weeks of refrigerated storage.

The present results clearly indicate that cow milk provides a more supportive environment for the proliferation and viability of starter cultures than goat milk. Despite a decline in LAB counts during storage, their levels in all samples were typical of yogurts characterized by high microbial quality. Therefore, cow–goat milk blends can be used to obtain yogurts with enhanced nutritional value and the required LAB counts, but the fermentation parameters and storage time of these products should be optimized.

The acidity (pH) of yogurts immediately after production ranged from 4.61 to 4.63, with no significant differences between the samples (*p* > 0.05), which indicates that the fermentation process was consistent and adequate in all milk blends. After two weeks of storage, a further decline in pH was noted in all samples, due to the metabolic activity of LAB and progressing acidification during refrigerated storage. The pH of yogurts was significantly (*p* < 0.05) affected by milk type and storage time, and a significant (*p* < 0.05) interaction between milk blend and storage time was also observed. These results indicate that the rate of changes in pH is determined by the proportions of cow milk and goat milk in yogurts. The lower ultimate pH values observed in goat milk yogurts may be related to differences in buffering behavior and the structural characteristics of goat milk casein micelles. However, it should be noted that literature reports on the buffering capacity of goat milk are inconsistent and may depend on factors such as goat breed and milk composition [32,33]. Previous studies have found that goat milk undergoes more pronounced acidification during the later stages of fermentation and subsequent storage, compared with cow milk [15,19].

### 3.3. Color

The color of milk and yogurts was analyzed instrumentally in CIE Lab* space, where parameter L* denotes lightness, a* is the contribution of red and green color components, b* is the contribution of yellow and blue color components, chroma (C*) is color saturation, and the whiteness index (WI*) measures how white a material appears (Table 3). The color analysis involved samples of cow milk and goat milk (A–E) as well as yogurt samples directly after production (A1–E1) and after two weeks of refrigerated storage (A3–E3).

The L* values of milk ranged from 86.60 (A—cow milk) to 85.94 (E—goat milk). These differences were significant (*p* < 0.05) and resulted from natural variations in the color of the analyzed raw materials. Goat milk appears to be whiter because it is less abundant in carotenoids, whereas β-carotene is responsible for the yellowish hue of cow milk [10,14]. A slight increase in L* values (87.44–87.42) was observed after fermentation, which may be attributed to light dispersion by the newly formed protein network and the homogenization of fat. Lightness values remained similar after storage, thereby confirming that the color of yogurt remains stable during refrigeration [21].

The values of a* were negative in all samples (−3.63 to −3.02), pointing to a higher contribution of green over red color components. The values of parameter a* increased with a rise in the proportion of goat milk, indicating reduced greenness and a shift toward warmer hues. Slight changes in a* values were observed after fermentation, but they remained stable during storage, which indicates that the fermentation process did not significantly affect this parameter.

Similar observations were made by Milovanovic et al. [34], who found that goat milk yogurts have a more neutral color than cow milk yogurts. According to Bulgaru et al. [15], these differences result from the absence of carotenoids in goat milk and a different fat profile that affects light dispersion in a product.

Parameter b* denotes the intensity of the yellow color, and it decreased with increasing proportions of goat milk—from 7.62 (A—cow milk) to 5.35 (E—goat milk). These differences were statistically significant (*p* < 0.05). A slight increase in b* values was observed after fermentation, which could be attributed to the concentration of milk components and optical changes induced by the formation of the protein gel. Domagała [18] and Mituniewicz-Małek et al. [23] found that goat milk products are characterized by a lower contribution of yellowness due to their low content of fat-soluble pigments and carotenoids, which corroborates the present findings.

Parameter C* is a measure of color intensity or saturation. In milk blends, C* values decreased with a rise in the proportion of goat milk—from 61.75 to 31.61. After fermentation, C* values increased in cow milk yogurts (79.50) and mixed-milk yogurts but remained lower in goat milk yogurts (49.01–43.62). These variations could result from differences in fat distribution and its impact on light dispersion in products [14]. The present findings are consistent with those of Sharma et al. [29] and Kandasamy et al. [30], who demonstrated that goat milk and its fermented products exhibit distinct profiles of volatile compounds and fatty acids, which also indirectly affect their perceived color and color intensity.

The WI* of milk ranged from 15.84 (A) to 15.35 (E) in milk and decreased to 15.60–14.59 in yogurts (A1–E1). This parameter was highest in cow milk yogurts and lowest in goat milk yogurts, which confirms that cow milk imparts a creamier hue to the product, whereas goat milk produces a whiter but less saturated color [1,10,35]. After storage, significant differences (*p* < 0.05) were observed between yogurt samples (A3–E3), but the overall variation in WI* values was low, pointing to the stability of yogurt color during refrigeration.

The color of milk and yogurt was significantly influenced by the proportion of goat milk. Yellowness (b*) and chroma (C*) decreased, whereas lightness (L*) and whiteness (WI*) increased slightly with a rise in the proportion of goat milk. These changes resulted from differences in carotenoid content, fat composition, and the degree of homogenization. Similar trends were described in previous studies, which have shown that the absence of β-carotene and the presence of smaller milk fat globules in goat milk result in products that are lighter, whiter, less yellow, and more milky in appearance [14,21,34].

### 3.4. WHC and Texture

The WHC of fresh yogurts ranged from 36.00% (A1) to 12.04% (E1). This parameter decreased significantly (*p* < 0.05) with increasing proportions of goat milk (Table 4). A further decrease in WHC values to 31.20–10.30% was observed after two weeks of storage. The greatest decline was noted in sample E3, suggesting that products with a predominance of goat milk are more susceptible to syneresis.

Firmness values followed a similar trend. Immediately after fermentation, this parameter was highest in sample A1 (2.04 N) and lowest in sample E1 made entirely from goat milk (0.39 N). After storage, firmness decreased in all samples, reaching 1.20–0.34 N. The observed decrease was significant (*p* < 0.05), and its magnitude increased with increasing proportions of goat milk. This finding suggests that casein gel in goat milk yogurts is weaker and less stable over time. A similar trend was also observed in consistency, which ranged from 25.61 N·s in yogurt A1 to 6.78 N·s in yogurt E1. After two weeks of storage, consistency decreased to 21.97–5.64 N·s. This decrease was correlated with the decline in firmness and WHC, confirming that the mechanical properties of yogurt are strongly linked to the gel’s ability to retain water. Cohesiveness, namely a gel’s ability to maintain its integrity, was determined at 0.53–0.39 N in fresh samples and at 0.48–0.35 N after storage. Cohesiveness was highest in sample A3 and lowest in sample E3. This parameter decreased significantly (*p* < 0.05) after storage, which points to the gradual structural rearrangements of the gel structure during storage.

The current results are consistent with published data. Dimitrellou et al. [21] reported that goat milk yogurts are characterized by lower firmness and lower WHC than cow milk yogurts due to differences in the structure of casein micelles and a lower content of κ-casein in goat milk. In turn, Mituniewicz-Małek et al. [27] found that the reduced buffering capacity of goat milk accelerates acidification, leading to the formation of gel with a looser structure and greater susceptibility to syneresis. According to Mani-López et al. [22] and Meybodi et al. [28], the textural properties of yogurt deteriorate during storage due to changes in pH and the protein network, and water migration from the gel matrix. The obtained results parallel these observations: after two weeks of storage, all analyzed textural attributes worsened significantly (*p* < 0.05), particularly in products with a high proportion of goat milk. Sharma et al. [29] and Kandasamy et al. [30] demonstrated that the presence of different metabolites in cow and goat milk yogurts, particularly free amino acids and medium-chain fatty acids, may affect gel stability via hydrophobic interactions and electrostatic forces between proteins. For this reason, products with higher proportions of goat milk have a less stable structure characterized by lower firmness and looser consistency. Bulgaru et al. [15] reported that goat milk yogurt has a looser and softer curd than cow milk yogurt due to differences in casein micelle structure and αs1-casein content. Goat milk yogurt was characterized by looser consistency, lower viscosity, and greater susceptibility to syneresis than cow milk yogurt. The properties of yogurt gel are influenced not only by milk type but also by additives, processing method and storage conditions. For example, Hovjecki et al. [36] demonstrated that the use of microbial transglutaminase (mTGase) in the production of goat milk yogurt increased gel firmness and cohesiveness, indicating that the texture of goat milk products can be improved through technological processes.

In conclusion, this study revealed that a higher proportion of goat milk in the raw milk blend significantly decreases the values of all textural parameters (WHC, firmness, consistency, and cohesiveness), both directly after production and after storage. These changes are linked to the specific protein and fat profile of goat milk as well as to post-fermentation processes during storage. The present findings indicate that in yogurts produced from mixed cow–goat milk, the proportion of cow milk should not be less than 50% to achieve the desired texture.

### 3.5. Analysis of Volatile Organic Compounds

**Figure 2:**
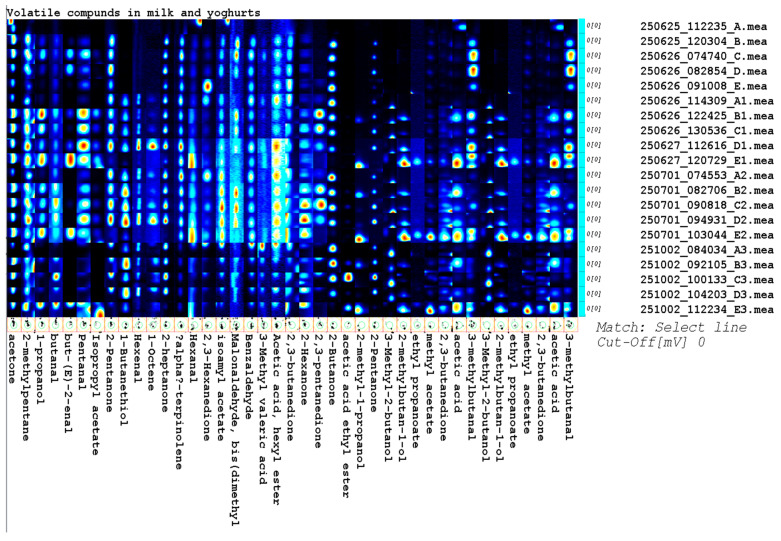
Heatmap of volatile organic compounds identified in milk and yogurts.

Volatile organic compounds (VOCs) were analyzed by chromatography coupled with electrochemical detection, and the results were presented in a heatmap (Figure 2). More than 40 volatile compounds belonging to the main chemical classes associated with the aroma of dairy products were identified, including aldehydes (hexanal, nonanal, 3-methylbutanal), ketones (2-heptanone, 2-nonanone, 2-pentanone), alcohols (1-propanol, 2-methyl-1-propanol, 3-methyl-1-butanol), esters (ethyl acetate, butyl acetate, ethyl propionate), acids (acetic acid, butyric acid), and terpenes (α-terpinolene). Pronounced differences in aromatic profiles were observed between raw milk samples (A–E) and yogurts at each stage of storage, with ketones, aldehydes, and esters generating the strongest signals in the post-fermentation samples. However, it should be noted that analytical signal intensity does not directly correspond to sensory impact, as many dairy aroma compounds exhibit very low odor perception thresholds.

Aldehydes, particularly hexanal, octanal, and pentanal, generated moderately strong signals in milk samples before fermentation (A–E), indicating a lipid oxidation profile typical of raw milk. These compounds are characterized by very low odor thresholds, which implies that even moderate analytical responses may translate into perceptible green, grassy, or fatty notes [37]. Ketones (2-heptanone and 2-nonanone) generated relatively weak signals, which reflects their predominantly microbial origin and the limited enzymatic activity in non-fermented milk; nevertheless, their low odor thresholds in water (0.001–0.01 mg·kg^−1^ for 2-heptanone and 0.041–0.082 mg·kg^−1^ for 2-nonanone) suggest that even small amounts may contribute to aroma perception [36]. In this group of samples, the weak signals generated by esters and alcohols point to the low natural metabolic activity of raw-milk microflora; however, their potential sensory contribution cannot be excluded due to their generally low odor perception thresholds.

The intensity of signals generated by compounds characteristic of LAB activity increased after production (A1–E1). Diacetyl (2,3-butanedione) and acetoin (3-hydroxy-2-butanone)—the metabolites responsible for the buttery and creamy aroma of yogurt—produced particularly strong signals; this observation is especially relevant from a sensory perspective, as diacetyl is known to have an extremely low odor threshold in water (approx. 0.0011 mg·kg^−1^) and can dominate aroma perception even at trace concentrations [37].

Esters, including ethyl acetate, butyl acetate, and ethyl propionate, were also identified, and their intensity was much higher than in raw milk samples, which reflects esterification processes occurring during fermentation; despite moderate signal intensities compared to other VOCs, these esters may strongly influence aroma due to their fruity character and low perception thresholds. Alcohols (1-propanol and 2-methyl-1-propanol), which are formed via the reduction of aldehydes under anaerobic conditions, also generated strong signals in this phase, contributing to the complexity of the fermented aroma.

The heatmap revealed further changes in the VOC profiles of yogurts after one week of storage (A2–E2). The greatest increase in signal intensity was observed for medium-chain ketones (2-heptanone and 2-nonanone) that are typically found in goat milk products as markers of lipolysis and β-oxidation; these compounds are known to possess low odor thresholds and impart characteristic pungent and fatty notes, suggesting a strong sensory impact even when the differences in concentration are relatively small. In this group of samples, strong signals were also produced by acetic and butyric acids that are responsible for sour and cheesy notes in yogurt during prolonged storage, particularly when their concentrations exceed sensory threshold values. The ester profile continued to expand, which indicates that LAB retained enzymatic activity even after the main fermentation phase. This suggests that LAB may progressively modulate aroma perception.

Yogurt samples analyzed on the last day of storage (A3–E3; the last items at the bottom of the heatmap) had the most complex and intense aromatic profile. Strong signals were generated by aldehydes (hexanal and 3-methylbutanal), ketones (2-heptanone and 2-nonanone), alcohols, and esters (ethyl acetate and esters of butyric and valeric acids). The consistently high intensity of ketones and medium-chain fatty acids is characteristic of goat milk products, reflecting their well-known tendency to generate compounds with a strong and pungent aroma, which is further amplified by their low odor perception thresholds. The accumulation of short-chain aldehydes (such as 3-methylbutanal) was also observed as storage progressed; these compounds are characterized by very low odor thresholds and are therefore considered highly aroma-active markers of amino acid (particularly leucine) transformations catalyzed by LAB enzymes.

Overall, the heatmap points to clear differences between milk and yogurt samples and between blends containing different proportions of cow milk and goat milk. Samples with higher proportions of goat milk showed stronger signals of ketones (2-heptanone and 2-nonanone), lipid-derived aldehydes, and medium-chain fatty acids, which collectively contribute to the characteristic aroma of goat milk, particularly due to the high sensory potency of these compounds. Notably, many of these volatiles are characterized by very low odor threshold values (e.g., 2-heptanone and short-chain aldehydes), which indicates that even relatively small changes in their concentration may result in pronounced sensory effects, including pungent, fatty, and animal-like notes typical of goat milk products.

In turn, cow milk samples showed stronger signals of esters and compounds responsible for mild, fruity, and creamy notes, whose sensory relevance is enhanced by their relatively low odor thresholds and pleasant aroma descriptors. Even when present at moderate concentrations, esters such as ethyl acetate and ethyl propionate may substantially influence overall aroma perception due to their high volatility and low perception thresholds, contributing to a smoother and more balanced flavor profile typical of cow-milk-based yogurts.

The observed differences in the VOC profiles of yogurts across successive storage days reflect the ongoing metabolic activity of LAB and the resulting development of yogurt aroma at both chemical and sensory levels. As highlighted in the literature, aroma perception during storage is driven not only by the accumulation of volatile compounds but also by shifts in the balance between low-threshold, aroma-active molecules derived from lipid oxidation, amino acid catabolism, and esterification processes, which together modulate the ultimate sensory character of yogurt.

The principal component analysis (PCA) enabled clear discrimination between milk and yogurt samples and revealed the relationships between their composition and VOC profiles (Figure S1). The first principal component (PC1; 33% of the variance) differentiated milk and yogurt samples regardless of storage day. Milk samples were clustered at negative PC1 values, whereas yogurt samples were shifted toward positive values, confirming the increased levels of ketones, esters, and fermentation-derived alcohols.

The second principal component (PC2; 24% of the variance) reflected the differences between raw milk blends. Blends with higher proportions of goat milk loaded toward aldehydes and medium-chain ketones, whereas those with a predominance of cow milk loaded toward esters associated with milder and fruity aroma notes. Yogurt samples also gradually diverged from the milk cluster, reflecting ongoing metabolic processes and the resulting development of aroma during storage.

The VOC profiles of yogurt samples reflect the previously described mechanisms of aroma formation in fermented products. A review by Alam et al. [37] highlights that the intensity and diversity of VOCs is determined by both the fermentation pattern and the properties of the raw materials, including lipid composition and the availability of substrates for LAB. These assumptions are consistent with the differences observed between the samples analyzed in this study. The proportion of goat milk was a particularly important factor, because its lipid profile promotes the formation of methyl ketones and aldehydes with a distinctive fatty aroma.

The current results also corroborate the findings of Bennato et al. [38], who demonstrated that free fatty acids and methyl ketones dominate in goat milk products as a result of more intense lipolysis and lipid autoxidation. In the present study, the highest concentrations of these compounds were observed in yogurts with the greatest proportions of goat milk, as reflected by sample positioning in PCA space. In turn, the higher ester content of yogurts made from cow milk suggests that different metabolic pathways predominate in this matrix. These compounds contribute mild, fruity, and creamy aroma notes, and their concentrations often increase during storage, which is consistent with both the heatmap and literature data.

A comparison of GC-IMS and PCA results with published findings indicates that the composition of raw materials and storage time are the key determinants of aroma development in yogurt. This study confirmed that cow–goat milk blends promote the formation of complex and characteristic VOC profiles, which are ultimately shaped by the interplay between lipid transformations and LAB metabolism.

### 3.6. Sensory Analysis

The mean values of the sensory scores of the analyzed yogurts, grouped into categories associated with appearance, aroma, consistency, taste, and overall acceptability, are presented in Table 5. All yogurts received very high scores for color uniformity (*p* > 0.05). The examined samples differed in the intensity of the creamy color, which was described as moderate in cow milk yogurt (A3) and yogurts made with 25% (B3) and 50% (C3) of goat milk. The intensity of the creamy color was evaluated as low in yogurts made with 75% (D3) and 100% goat milk (E3). Whey separation followed an opposite trend and increased significantly (*p* < 0.05) with a rise in the proportion of goat milk in the analyzed products, which is typical of goat milk yogurt [39,40].

Aroma characteristics were assessed at the next stage of the sensory analysis. The analyzed yogurts differed significantly (*p* < 0.05) in the intensity of yogurt-like, sour, and goaty aromas. Yogurt made from 100% cow milk had a very strong yogurt aroma. The yogurt-like aroma was evaluated as intense in yogurts made with 25% and 50% goat milk, and as moderate in yogurts made with 75% and 100% goat’s milk. In turn, the intensity of the sour aroma increased gradually and was described as low in yogurts A3 and B3 and as moderate in yogurts C3, D3, and E3. The intensity of the goaty aroma increased with a rise in the proportion of goat milk and was evaluated as very low and low in yogurts made from cow–goat milk blends and as high in goat milk yogurt. No atypical aroma was detected in any of the analyzed yogurts.

The analysis of consistency attributes revealed significant differences (*p* < 0.05) between the samples in terms of uniformity, thickness, and smooth mouthfeel. The uniformity of cow milk yogurt (A3) was evaluated as very strong. Consistency was described as highly uniform in yogurt made with 25% goat milk (B3) and as moderately uniform in the remaining products (C3, D3, E3). Moreover, cow milk yogurt (A3) was evaluated as moderately thick and received the highest score for thickness among the tested products. Yogurts B3 and C3 were characterized by low thickness, whereas yogurts made with 75% (D3) of goat milk and produced from 100% goat milk (E3) were described as very thin. These results indicate that yogurt thickness decreased with a rise in the proportion of goat milk. The mouthfeel of yogurts was rated as very smooth (A3) and smooth (B3, C3, D3, and E3).

The analyzed yogurts differed in the intensity of yogurt-like, sour, goaty, and salty tastes (*p* < 0.05). The yogurt taste was evaluated similarly to the yogurt aroma. Yogurt made from 100% cow milk (A3) exhibited a very strong yogurt-like taste. The intensity of the yogurt-like taste was described as high in yogurts made with 25% and 50% goat milk and as moderate in those produced with 75% proportion and 100% goat milk. The sour taste was described as strong in yogurt made with 100% goat milk (E3), moderate in yogurts C3 and D3, and weak in yogurts with the highest content of cow milk (A3 and B3). In turn, the goaty taste was very strong in goat milk yogurt (E3) and strong in yogurt made with 75% goat milk (D3). The intensity of the goaty taste declined rapidly and was described as weakly discernible when the proportion of goat milk decreased below 50% (C3). The goaty taste was not detected in cow milk yogurt. The salty taste was barely detectable in yogurts A3, B3, and C3, and it was described as very weak in yogurts with the highest proportions of goat milk. No atypical taste was detected in any of the analyzed yogurts.

The overall acceptability of yogurts was evaluated at the last stage of the sensory analysis. Cow milk yogurt received the highest score in this regard. In the group of yogurts containing goat milk, the product made with 25% proportion of goat milk received the highest score for overall acceptability. This sample was characterized by the lowest syneresis, a strong yogurt-like taste and aroma, a very weak goaty aroma and taste, and a mild sour taste comparable to that of cow milk yogurt.

## 4. Summary and Conclusions

Milk type influenced the fermentation pattern and the attributes of the produced yogurts. An analysis of acidification curves revealed that pH decreased at a slower rate in yogurts with higher proportions of cow milk. These yogurts were characterized by more desirable technological properties, greater cohesiveness, and higher LAB survival rates. Increasing the proportion of cow milk improved textural attributes and reduced syneresis, which can be attributed to differences in casein structure and the lower buffering capacity of goat milk. Yogurts with higher proportions of goat milk were characterized by a lighter color, lower intensity of the creamy color, and greater whiteness. These samples exhibited a predominance of free fatty acids and methyl ketones as a result of more intense lipolysis and lipid autoxidation. The highest concentrations of these compounds were found in yogurts with the greatest proportions of goat milk.

The sensory analysis confirmed that the intensity of the creamy color decreased, whereas whey separation increased with a rise in the proportion of goat milk in the tested yogurts. It should be noted that increasing the proportion of goat milk reduced consistency uniformity to a moderate level and significantly decreased thickness, while maintaining a very smooth mouthfeel. In addition, increasing the proportion of goat milk did not completely diminish the intensity of yogurt-like aroma and taste, which should be regarded as an advantage. Sour and goaty tastes were most discernible in yogurts with the highest proportions of goat milk. Cow–goat milk blends may offer a favorable compromise, combining acceptable texture and stability with the higher nutritional value and distinct sensory profile contributed by goat milk.

## Figures and Tables

**Table 1:** Chemical composition of milk and yogurts.

Parameter	Milk					Yogurt				
	A	B	C	D	E	A1	B1	C1	D1	E1
Fat (%)	4.29 ± 0.00 ^a^	4.34 ± 0.00 ^b^	4.43 ± 0.00 ^c^	4.47 ± 0.01 ^d^	4.55 ± 0.00 ^e^	4.21 ± 0.02 ^a^	4.27 ± 0.04 ^b^	4.36 ± 0.02 ^c^	4.41 ± 0.03 ^d^	4.48 ± 0.02 ^e^
Total protein (%)	4.19 ± 0.01 ^a^	4.19 ± 0.01 ^a^	4.16 ± 0.01 ^b^	4.15 ± 0.02 ^b^	4.14 ± 0.02 ^b^	4.15 ± 0.01 ^a^	4.07 ± 0.02 ^c^	4.08 ± 0.01 ^bc^	4.09 ± 0.02 ^bc^	4.10 ± 0.02 ^b^
Lactose (%)	6.06 ± 0.01 ^a^	5.95 ± 0.01 ^b^	5.86 ± 0.01 ^c^	5.79 ± 0.01 ^d^	5.69 ± 0.01 ^e^	5.69 ± 0.03 ^a^	5.57 ± 0.04 ^b^	5.46 ± 0.07 ^c^	5.30 ± 0.03 ^d^	5.13 ± 0.01 ^e^
Dry matter (%)	15.40 ± 0.01 ^a^	15.34 ± 0.01 ^b^	15.29 ± 0.01 ^c^	15.08 ± 0.01 ^d^	14.95 ± 0.00 ^e^	15.31 ± 0.05 ^a^	15.19 ± 0.04 ^b^	15.08 ± 0.02 ^c^	14.92 ± 0.03 ^d^	14.74 ± 0.01 ^e^

Means ± standard deviation (*n* = 3); ^a,b,c,d,e^—Mean values marked with different letters in the same row differ significantly at *p* < 0.05; A, B, C, D, E—control milk; A1, B1, C1, D1, E1—yogurts directly after production.

**Table 2:** Starter culture counts and active acidity in yogurts.

Yogurt		*Lactobacillus delbrueckii* ssp. *bulgaricus* (log CFU/mL)	*Streptococcus thermophilus* (log CFU/mL)	pH
Directly after production	A1	7.24 ± 0.10 ^a^	8.79 ± 0.04 ^a^	4.63 ^a^
	B1	7.23 ± 0.03 ^a^	8.74 ± 0.11 ^ab^	4.63 ^a^
	C1	7.14 ± 0.05 ^a^	8.69 ± 0.04 ^ab^	4.61 ^a^
	D1	6.83 ± 0.13 ^b^	8.64 ± 0.02 ^ab^	4.62 ^a^
	E1	6.71 ± 0.09 ^bc^	8.59 ± 0.16 ^bc^	4.62 ^a^
After 2 weeks of storage	A3	6.67 ± 0.12 ^bc^	8.44 ± 0.03 ^cd^	4.44 ^b^
	B3	6.53 ± 0.12 ^cd^	8.40 ± 0.02 ^d^	4.36 ^c^
	C3	6.44 ± 0.11 ^de^	8.34 ± 0.04 ^de^	4.34 ^c^
	D3	6.26 ± 0.1 ^e^	8.21 ± 0.02 ^e^	4.24 ^d^
	E3	6.01 ± 0.05 ^f^	8.01 ± 0.09 ^f^	4.13 ^e^
Significance (*p* value)	M	0.000	0.000	0.000
	S	0.000	0.000	0.000
	m × s	NS	NS	0.000

Means ± standard deviation (n = 3); m, mixture; s, storage; m × s, mixture and storage interaction; ^a,b,c,d,e,f^—Mean values marked with different letters in the same column differ significantly at *p* < 0.05; A1, B1, C1, D1, E1—yogurts directly after production, A3, B3, C3, D3, E3—yogurts after 2 weeks of storage; NS—not significant.

**Table 3:** Color parameters of milk and yogurts.

Milk		L*	a*	b*	C*	IW*
Control milk	A	86.60 ± 0.03 ^a^	−3.63 ± 0.01 ^e^	7.62 ± 0.01 ^a^	61.75 ± 0.09 ^a^	15.84 ± 0.02 ^a^
	B	86.44 ± 0.05 ^b^	−3.52 ± 0.01 ^d^	7.07 ± 0.01 ^b^	53.56 ± 0.08 ^b^	15.69 ± 0.04 ^b^
	C	86.20 ± 0.02 ^c^	−3.33 ± 0.02 ^c^	6.58 ± 0.01 ^c^	46.63 ± 0.12 ^c^	15.65 ± 0.10 ^b^
	D	86.12 ± 0.02 ^c^	−3.21 ± 0.01 ^b^	6.02 ± 0.01 ^d^	39.41 ± 0.08 ^d^	15.46 ± 0.02 ^c^
	E	85.94 ± 0.02 ^d^	−3.02 ± 0.01 ^a^	5.35 ± 0.01 ^e^	31.61 ± 0.05 ^e^	15.35 ± 0.02 ^d^
**Yogurt**						
Directly after production	A1	87.44 ± 0.02 ^cd^	−3.00 ± 0.01 ^e^	8.75 ± 0.01 ^d^	79.50 ± 0.19 ^d^	15.60 ± 0.02 ^ab^
	B1	87.50 ± 0.03 ^c^	−3.13 ± 0.02 ^g^	8.84 ± 0.01 ^c^	81.28 ± 0.16 ^c^	15.63 ± 0.02 ^a^
	C1	86.99 ± 0.04 ^e^	−2.97 ± 0.01 ^d^	7.50 ± 0.00 ^f^	59.22 ± 0.01 ^f^	15.31 ± 0.03 ^c^
	D1	86.93 ± 0.07 ^e^	−2.85 ± 0.02 ^a^	6.49 ± 0.01 ^i^	44.97 ± 0.13 ^i^	14.87 ± 0.06 ^e^
	E1	87.42 ± 0.04 ^cd^	−2.91± 0.01 ^b^	6.79 ± 0.01 ^g^	49.01 ± 0.13 ^g^	14.59 ± 0.03 ^f^
After 2 weeks of storage	A3	87.65 ± 0.14 ^b^	−3.12 ± 0.02 ^g^	8.88 ± 0.01 ^b^	81.92 ± 0.20 ^b^	15.53 ± 0.11 ^b^
	B3	87.95 ± 0.02 ^a^	−3.20 ± 0.02 ^h^	9.02 ± 0.01 ^a^	84.62 ± 0.12 ^a^	15.39 ± 0.02 ^c^
	C3	87.50 ± 0.01 ^c^	−3.06 ± 0.01 ^f^	7.65 ± 0.01 ^e^	61.53 ± 0.09 ^e^	14.97 ± 0.01 ^d^
	D3	87.39 ± 0.05 ^d^	−2.94 ± 0.02 ^c^	6.66 ± 0.00 ^h^	47.30 ± 0.02 ^h^	14.56 ± 0.05 ^f^
	E3	87.38 ± 0.04 ^d^	−3.04 ± 0.01 ^f^	6.37 ± 0.06 ^j^	43.62 ± 0.79 ^j^	14.46 ± 0.06 ^g^
Significance (*p* value)	m	0.000	0.000	0.000	0.000	0.000
	s	0.000	0.000	0.000	0.000	0.000
	m × s	0.000	0.006	0.000	0.000	0.001

Means ± standard deviation (n = 3); m, mixture; s, storage; m × s, mixture and storage interaction; ^a,b,c,d,e,f,g,h,i,j^—Mean values marked with different letters in the same column differ significantly at *p* < 0.05; A, B, C, D, E—control milk; A1, B1, C1, D1, E1—yogurts directly after production, A3, B3, C3, D3, E3—yogurts after 2 weeks of storage.

**Table 4:** Textural properties of yogurts.

Yogurt		WHC (%)	Firmness (N)	Consistency (N s)	Cohesiveness (N)
Directly after production	A1	36.00 ± 2.55 ^a^	2.04 ± 0.13 ^a^	25.61 ± 2.14 ^a^	0.53 ± 0.01 ^a^
	B1	30.63 ± 0.86 ^b^	1.46 ± 0.06 ^b^	21.41 ± 0.59 ^b^	0.49 ± 0.03 ^ab^
	C1	25.70 ± 0.93 ^c^	0.90 ± 0.08 ^d^	17.67 ± 0.75 ^c^	0.45 ± 0.01 ^cd^
	D1	19.55 ± 0.40 ^e^	0.64 ± 0.06 ^e^	13.12 ± 0.35 ^e^	0.38 ± 0.01 ^ef^
	E1	12.04 ± 0.38 ^g^	0.39 ± 0.02 ^g^	6.78 ± 0.61 ^g^	0.39 ± 0.04 ^e^
After 2 weeks of storage	A3	31.20 ± 1.62 ^b^	1.20 ± 0.03 ^c^	21.97 ± 1.42 ^b^	0.48 ± 0.02 ^bc^
	B3	26.29 ± 2.33 ^c^	1.12 ± 0.07 ^c^	18.16 ± 1.86 ^c^	0.45 ± 0.01 ^cd^
	C3	22.33 ± 0.28 ^d^	0.71 ± 0.05 ^e^	15.00 ± 0.21 ^d^	0.43 ± 0.01 ^d^
	D3	14.94 ± 0.51 ^f^	0.51 ± 0.06 ^f^	9.59 ± 0.30 ^f^	0.34 ± 0.02 ^g^
	E3	10.30 ± 0.68 ^g^	0.34 ± 0.02 ^g^	5.64 ± 0.36 ^g^	0.35 ± 0.03 ^fg^
Significance (*p* value)	m	0.000	0.000	0.000	0.000
	s	0.000	0.000	0.000	0.000
	m × s	NS	0.000	NS	NS

Means ± standard deviation (n = 3); m, mixture; s, storage; m × s, mixture and storage interaction; ^a,b,c,d,e,f,g^—Mean values marked with different letters in the same column differ significantly at *p* < 0.05; A1, B1, C1, D1, E1—yogurts directly after production, A3, B3, C3, D3, E3—yogurts after 2 weeks of storage; NS—not significant; WHC—water-holding capacity.

**Table 5:** Mean values of the sensory scores of yogurts made from cow milk, goat milk, and mixed cow–goat milk directly after production (n = 8).

SENSORY ATTRIBUTES	A3	B3	C3	D3	E3	*p*-Value
APPEARANCE						
Color uniformity	6.6	6.5	6.5	6.5	6.6	>0.05
Creamy color	4.7 ^a^	4.7 ^a^	4.1 ^b^	3.5 ^c^	3.5 ^c^	0.000
Whey syneresis	1.0 ^e^	1.9 ^d^	2.4 ^c^	3.3 ^b^	5.5 ^a^	0.000
AROMA						
Yogurt-like	6.5 ^a^	5.7 ^b^	5.5 ^b^	4.9 ^c^	4.6 ^c^	0.000
Sour	3.8 ^c^	3.9 ^c^	4.3 ^b^	4.6 ^a^	4.8 ^a^	0.002
Goaty	1.0 ^d^	2.3 ^c^	2.9 ^b^	3.0 ^b^	5.0 ^a^	0.000
Atypical	1.0	1.0	1.0	1.0	1.0	>0.05
CONSISTENCY						
Uniform	6.6 ^a^	5.4 ^b^	4.7 ^c^	4.6 ^c^	4.3 ^c^	0.000
Thick	4.6 ^a^	3.7 ^b^	3.6 ^b^	2.6 ^c^	2.5 ^c^	0.000
Smooth mouthfeel	6.1 ^a^	5.6 ^b^	5.6 ^b^	5.5 ^b^	5.5 ^b^	0.000
TASTE						
Yogurt-like	6.8 ^a^	5.8 ^b^	5.5 ^b^	4.8 ^c^	4.5 ^c^	0.005
Sour	3.7 ^c^	3.9 ^c^	4.7 ^b^	4.8 ^b^	5.3 ^a^	0.000
Goaty	1.0 ^e^	2.5 ^d^	3.2 ^c^	5.1 ^b^	6.2 ^a^	0.000
Salty	1.1 ^b^	1.2 ^b^	1.2 ^b^	2.6 ^a^	2.9 ^a^	0.000
Atypical	1.0	1.0	1.0	1.0	1.0	>0.05
OVERALL ACCEPTABILITY	6.1 ^a^	5.3 ^b^	4.7 ^c^	4.4 ^c^	3.5 ^d^	0.000

^a,b,c,d,^^e^–Mean values marked with different letters in the same row differ significantly at *p* < 0.05; A3—yogurt made from 100% cow milk, after 2 weeks of storage; B3—yogurt made from 75% cow milk + 25% goat milk, after 2 weeks of storage; C3—yogurt made from 50% cow milk + 50% goat milk, after 2 weeks of storage; D3—yogurt made from 25% cow milk + 75% goat milk, after 2 weeks of storage; E3—yogurt made from 100% goat milk, after 2 weeks of storage.

## Data Availability

The original contributions presented in this study are included in the article/Supplementary Materials. Further inquiries can be directed to the corresponding author.

## Supplementary Materials

The following supporting information can be downloaded at: https://www.mdpi.com/article/10.3390/foods15020314/s1, Figure S1. Principal component analysis (PCA) biplot of volatile components in milk and yogurts.
