# Supplementary figures and images for: Evaluation of the Fermentation Profiles and Quality Attributes of Yogurts Made from Cow, Goat, and Mixed Milk
Source: Foods. 2026 Jan 15;15(2):314. doi: 10.3390/foods15020314 (PMC12841329; doi:10.3390/foods15020314)

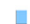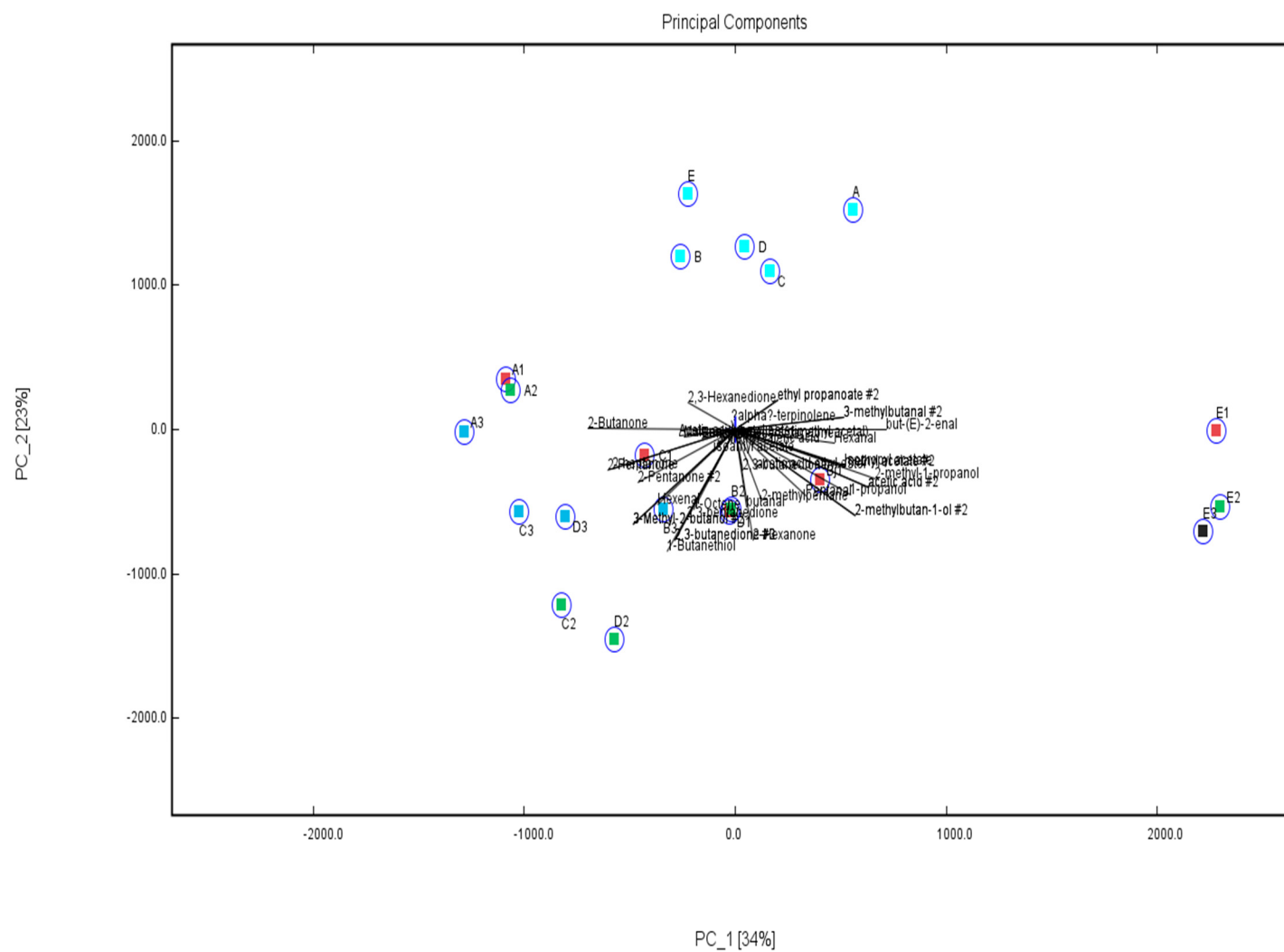

**Figure S1.** Principal component analysis (PCA) biplot of volatile components in milk and yogurts

Supplement: Supplementary file 1 [file foods-15-00314-s001.zip › foods-4079267-supplementary.pdf]
